# The effect of static scanning and mobility training on mobility in people with hemianopia after stroke: A randomized controlled trial comparing standardized versus non-standardized treatment protocols

**DOI:** 10.1186/1471-2377-11-87

**Published:** 2011-07-19

**Authors:** Stacey George, Allison Hayes, Celia Chen, Maria Crotty

**Affiliations:** 1 Flinders University, Division of Rehabilitation, Aged Care and Allied Health, Repatriation General Hospital, Daw Park, South Australia, 5043, Australia; 2 Neuro Vision Technology, PO Box 141, Torrensville, South Australia, 5031, Australia; 3 Department of Ophthalmology, Flinders Medical Centre and Flinders University, Flinders Drive, Bedford Park, South Australia, 5042 Australia

## Abstract

**Background:**

Visual loss following stroke impacts significantly on activities of daily living and is an independent risk factor for becoming dependent. Routinely, allied health clinicians provide training for visual field loss, mainly with eye movement based therapy. The effectiveness of the compensatory approach to rehabilitation remains inconclusive largely due to difficulty in validating functional outcome with the varied type and dosage of therapy received by an individual patient. This study aims to determine which treatment is more effective, a standardized approach or individualized therapy in patients with homonymous hemianopia post stroke.

**Methods/Design:**

This study is a double-blind randomized controlled, multicenter trial. A standardised scanning rehabilitation program (Neuro Vision Technology (NVT) program) of 7 weeks at 3 times per week, is compared to individualized therapy recommended by clinicians.

**Discussion:**

The results of the trial will provide information that could potentially inform the allocation of resources in visual rehabilitation post stroke.

**Trial Registration:**

Australia and New Zealand Clinical Trials Register (ANZCTR): ACTRN12610000494033

## Background

Stroke is a leading cause of disability [1], and approximately 30-35% of people with stroke have a homonymous hemianopia [2]. Hemianopia is described as "blindness in one half the field of vision in one or both eyes" [3]. Literature suggests that natural recovery occurs in 50% of people, mostly in the first month with minimal recovery taking place after 6 months [2]. Visual loss, following a stroke can be temporary or permanent, and interfere with an individual's ability to perform daily living activities and live independently [4].

Visual training in homonymous hemianopia post stroke is recognized as an important part of rehabilitation [5]. The project described in this manuscript is comparing the effects of two approaches to treating visual field loss in the subacute rehabilitation phase (week two - six months) following a stroke. Currently rehabilitation employs three key strategies including optical devices, compensatory training and visual restitution therapy [6]. The first strategy is to use optical devices, such as prism glasses, to displace the visual image. The efficacy of prisms has not been demonstrated due to the lacking of a standardized methodology and few controlled studies [7].

The second strategy is compensatory and aimed at enhancing eye movements and/or head movements. This takes a number of different forms including the training of oculomotor or saccadic movements, on a standardised computer screen, or devices such as the Dynavision [8], which incorporates a larger visual area, where head movements are required. Oculomotor scanning performance, thus only requiring eye movement, has been linked to subjects reported visual difficulties in everyday life [9]. Training of oculomotor or saccadic movements has been demonstrated to lead to an improved visual search field and reading ability [10-12]. No large scale randomized controlled trials have been performed to date to validate this intervention [13]. Dynavision training has been demonstrated to lead to improvement in visual scanning in a single case study [14], and in a descriptive study of driving [15], but not in a small controlled study of driving in stroke [16].

The third strategy uses visual restitution therapy and aims to enhance neuroplasticity [17]. This method involves the stimulation of the 'borderzone' or 'transition zone' between the area of visual loss and the intact area of vision [18]. A systematic review found [13] inconclusive evidence to support visual restitution based therapy as a means of restoring visual fields following brain damage.

In Australia, most visual training employs compensatory eye and head movement based therapy but current delivery of vision rehabilitation is recommended by treating therapists and is thus subjective [19]. Therapies can vary from a comprehensive combined static and mobility training to the prescription of magnifiers to help with reading with minimal instruction of their use. Due to the wide variability in type and dosage of therapy received by an individual patient, the benefits of visual training in terms of compensation and improved performance in functional tasks are uncertain [20].

**Figure 1:**
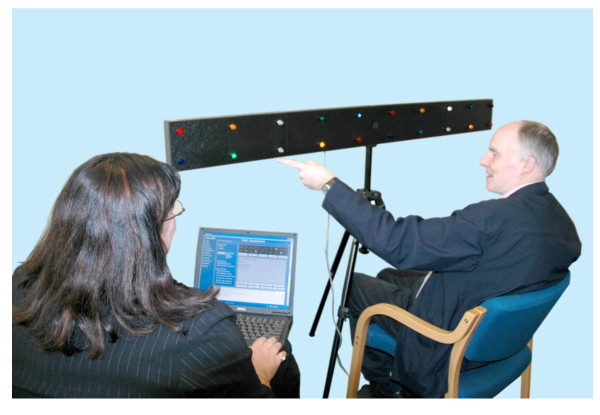
**NVT Scanning Device**.

In response to the need for a structured therapy program of visual training in homonymous hemianopia post stroke, the Neuro Vision Technology (NVT) program has been developed which is a standardized therapy and involves both a static scanning training and a dynamic mobility component. The static scanning component uses the NVT device, (see figure 1) to assess for the presence of neurological vision impairment, such as a homonymous hemianopia and/or visual spatial neglect. A standardized presentation of colored lights (LEDs) is displayed on a horizontal panel, placed at eye level approximately 30 cms from the patient. The panel of lights extends approximately 80 cms either side of central fixation, with the lights at the ends of the panel subtending an angle of approximately 70 degrees from midline. In the presence of a homonymous hemianopia the patient is required to use both head movement and eye movement towards the affected visual field to detect the light stimulus.

The NVT scanning device is used in rehabilitation to teach a pattern of systematic visual search strategies from the perimeter of the affected visual field towards the intact visual field. It aims to improve the patient's awareness and understanding of the nature of their vision impairment. The NVT training program then transfers visual scanning strategies, established using the NVT scanning device, into tasks that are mobility based. This developed NVT training differs from the other compensatory techniques as it involves a larger real visual world, whereas other compensatory training is aimed at rehabilitating reading abilities using a computer screen. NVT training has not been evaluated in terms of its effectiveness in rehabilitation for people with hemianopia.

To determine the most effective rehabilitation for people who have a hemianopia after stroke, it is necessary to examine the effects of the standardized and non-standardized rehabilitation strategies on performance in daily activities. We aim to compare the effectiveness of the standardized NVT program versus current individualized therapy as recommended by treating clinicians in patients with homonymous hemianopia post stroke.

## Methods and design

**Figure 2:**
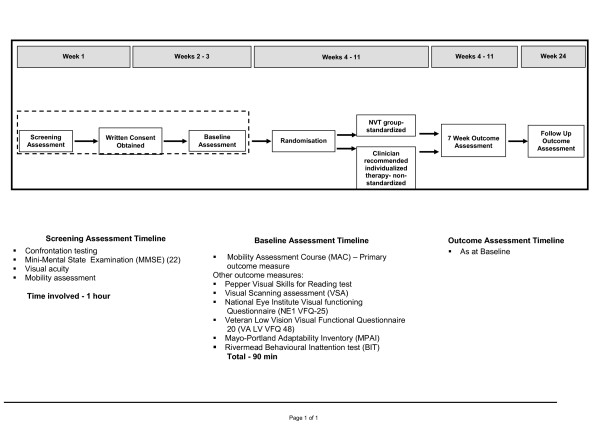
**Study Design**.

The study design is a single-blind randomized, controlled trial in Adelaide, Australia, which will be conducted over 12 months. The intervention design is summarised in Figure 2.

### Randomized controlled trial

#### Participants

Patients will be included if they have had a stroke occurring a minimum of two weeks and a maximum of six months prior to the commencement of assessment; homonymous hemianopia (any field loss reached above and below the horizontal axis, as measured by Medmont C-100 perimetry [21] performed by a neurophthalmologist); score of 25 or more on the Mini Mental State Examination (MMSE) [22]; have corrected vision of at least 6/18; be able to mobilise a distance of 35 metres independently or with supervision or standby assistance; and be over 18 years of age. Formal assessment related to the inclusion criteria is detailed in Figure 2.

Patients will be excluded based on evidence of aphasia or poor English language skills that significantly impact on understanding instructions.

#### Setting/Locations

Participants will be recruited from acute hospitals, rehabilitation hospitals and outpatient settings in Adelaide, Australia.

#### Procedures/Measurements

All outcome assessments will take place face to face with participants by assessors who are blind to the allocation group. All outcome assessments (listed in Figure 2) will be performed by mobility instructors in vision rehabilitation who have received training in the standardized use of the tools.

#### Recruitment

Participants will be recruited from all three publicly funded rehabilitation facilities in Adelaide. In addition we will recruit from private rehabilitation facilities, neurophthalmologists and the two non government organisations who provide training for visual impairment in South Australia, Australia.

#### Randomization

Randomization is managed by the pharmacy department at the Repatriation General Hospital. A statistician external to the study will generate the random sequence using the random number generator in Microsoft Excel and created sequentially numbered, sealed envelopes containing group allocation for participants.

#### Intervention 1

Those participants who are randomized to the intervention group 1 will receive the standardized NVT program that consist of: three weeks static scanning training using the NVT scanning device and four weeks of mobility training using NVT scanning techniques.

All assessment and training tasks using the NVT scanning device are standardized using the Vision 2000 software program. Patient responses to the light stimulus are also recorded in a manner that allows for uniformity of performance measurement. The assessment process initially uses single light presentations to determine the patient's degree of spontaneous scanning into the affected field, and the degree of visual field loss. Subsequent light sequences utilize multiple lights in exercises, which are graded in complexity. Patient responses to these exercises are indicative of their ability to attend to multiple stimuli, their ability to adopt a systematic search pattern, the speed at which visual stimuli can be detected and the ability of the patient to utilize spatial reasoning to compare light patterns. The NVT assessment on the scanning device informs the NVT training process.

The NVT scanning device is used to teach a pattern of systematic visual search strategies from the perimeter of the affected visual field towards the intact visual field. These training exercises utilize patterns of lights that reinforce an efficient and functional speed of scanning to allow for accurate detection of all stimuli. Refer to Figure 1.

The NVT training program then transfers visual scanning strategies, established using the NVT scanning device, for example the amount of head turning and frequency, into the real world while walking, with the aim being for these strategies to become automatic. Scanning skills are graded from an environment that is structured and familiar to the patient, into more dynamic busy environments including shopping centres, where high level scanning skills and complex visual processing is required. Training will occur three times per week for 7 weeks. Audiovisual information of the intervention and NVT device is accessible through the following link: http://www.scivee.tv/node/16116.

#### Intervention group 2

Participants in intervention group 2 will receive individualized, or non-standardized therapy recommended by clinicians or usual care. "Usual care" for people receiving rehabilitation after stroke varies across inpatient and ambulatory rehabilitation units. However, intervention currently available includes one to one occupational therapy and mobility instruction that promotes visual scanning, activity of daily living skills, and mobility training from a non government organization, separate from health services, the Guide Dogs Association SA.NT. Techniques for intervention include pen and paper tasks, scanning training on light boards, compensatory training in activities of daily living and mobility. Dosage and type of therapy will be determined by the treating clinician and recorded. This group will be wait-listed for the NVT training which will be offered at the completion of the three month follow up.

#### Outcomes

As walking ability is a skill which is valued by those who have suffered a stroke and is required for safe discharge from hospital our primary outcome will be performance on the Mobility Assessment Course (MAC) [23] after three months. The MAC assesses performance on a mobility course with targets positioned on the walls along the course. Participants are scored on the number of targets they identify and the time taken to complete the course. The MAC, along with the Visual scanning analyzer (VSA) [23], were designed to test visual scanning. The MAC measures the extent to which a person visually scans and identifies hazards when walking, and has been validated with stroke patients [23]. In this validation study [23] the MAC included a measure of obstacle avoidance, which mobility evaluations generally include, however the results concluded that this was a poor measure of visual neglect in this population and thus was not used in MAC data collection in this study protocol.

The secondary outcome assessments will provide a broader picture of function and quality of life following a stroke and include Visual scanning analyzer (VSA) [23], Pepper Visual Skills for Reading test [24], National Eye Institute Visual functioning Questionnaire (NE1 VFQ-25) [25], Veteran Low Vision Visual Functional Questionnaire 20 (VA LV VFQ 48) [26], Mayo-Portland Adaptability Inventory (MPAI) [27] and the Rivermead Behavioural Inattention test (BIT) [28] as listed in Figure 2.

#### Sample size

Sample size has been calculated based on data reported in a pilot study validating the primary outcome measurement tool, the Mobility Assessment Course [23]. The minimum number of participants required is 20, with 10 in each of the two groups providing 80% power (alpha level 0.05) to detect a mean difference 0.63 on scores, assuming the standard deviation is 0.74. Analysis of the results is planned in 2010 and will be undertaken by statisticians blind to the group allocation.

#### Statistical analysis

Data will be entered into an SPSS database with all identifying details removed. An ANCOVA will be performed to compare outcomes between the intervention groups, controlling for initial scores as covariates in the analyses.

Statistical analyses will be undertaken using SPSS version 17.0 Statistical software. All analyses will on an intention to treat basis and will be blinded (i.e. groups identified by number only) and results published according to the Consort Statement formal.

## Discussion

Visual rehabilitation training is routinely provided for people with hemianopia following stroke however the benefits of visual rehabilitation training on functional outcomes have not been well evaluated. Despite us knowing it is important to rehabilitation as it impacts negatively on independence following stroke [4].

The goal of visual rehabilitation involves making the most of a person's residual vision, specifically to try to overcome the visual disabilities that are most troublesome to them. Part of the difficulty in evaluating the effectiveness of vision rehabilitation in homonymous hemianopia is the varied type of strategies used by therapists. At the moment the details of the rehabilitation program are negotiated between each patient and their rehabilitation therapist. No robust evidence currently exists to identify the most effective way to deliver vision rehabilitation in people with homonymous hemianopia after a stroke or the optimum time required [13].

There is evidence that providing more exercise therapy early after stroke improves peoples' ability to recover [29] but the effects of additional therapy in vision rehabilitation are unclear. No large scale randomized controlled trials have been performed to establish the effectiveness of visual rehabilitation following brain damage [13].

This project will compare the effectiveness of current visual rehabilitation protocols which vary in type and dosage according to individual clinicians' recommendations, to a new standardized technology (NVT). Functional outcome measures, such as performance on a mobility assessment course and scanning ability will be employed as well as patient reported outcomes such as, vision specific health related quality of life to allow a meaningful assessment of this rehabilitation intervention.

A debate exists around the mechanisms which underlie the remedial approach in visual rehabilitation following stroke, that is compensatory or neuroplasticity [7]. The outcome of our proposed study will contribute to the knowledge of the mechanisms behind the compensatory approaches, currently used in clinical practice. Furthermore, the results of the trial will provide information that will inform the allocation of resources in visual rehabilitation post stroke. This is in terms of dosage and type of therapy provided.

As in many areas of rehabilitation more rigorous research is needed to identify which remedial approaches should be included in clinical practice for visual loss following stroke.

## Competing interests

Allison Hayes has participated in the development of the device and currently involved in the commercialisation.

## Authors' contributions

All authors contributed to the design and conduct of the clinical trial. SG carried out data analysis, and drafted the manuscript. AH conceived of the study and participated in its design and coordination. AD and MC participated and oversees the trial process. CC is involved in the neuro-ophthalmic assessment and data acquisition of all participants and helped draft the manuscript. All authors read and approved the final manuscript.

Written informed consent was obtained from the patient for publication of this case report and accompanying images. A copy of the written consent is available for review by the Editor-in-Chief of this journal.

## Pre-publication history

The pre-publication history for this paper can be accessed here:

http://www.biomedcentral.com/1471-2377/11/87/prepub

## References

[B1] National Stroke Foundation Facts and Figures http://www.strokefoundation.com.au/facts-figures-and-stats Accessed 11/9/08

[B2] Zhang X Kedar S Lynn M Newman N Biousse B Natural history of homonymous hemianopia Neurology 2006 66 901 905 10.1212/01.wnl.0000203338.54323.22 16567709

[B3] Dorland's Illustrated Medical Dictionary 1957 23 Philadelphia, W.B. Saunders Company 599

[B4] Denes G Semanza C Stoppa E Lis A Unilateral spatial neglect and recovery from hemiplegia Brain 1982 105 543 552 10.1093/brain/105.3.543 7104665

[B5] Collopy D Petherick M Clarke G A team approach to managing a patient with a neurological vision impairment Journal of the Australasian Rehabilitation Nurses Association 2001 4 16 20

[B6] Roman JG Progress in rehabilitation in hemianopic visual field defects Cerebrovas Dis 2009 27 187 190 19342850 10.1159/000200458

[B7] Pelak VS Dubin M Whitney E Homonymous hemianopia: a critical analysis of optical devices, compensatory training, and novavision Current Treatment Options in Neurology 2007 9 41 47 10.1007/s11940-007-0029-y 17288888

[B8] Klavora P Warren M Leung M Dynavision for rehabilitation of visual and motor deficits: A user's guide Birmingham: visABILITIES Rehab Services Inc 1996

[B9] Zihl J Oculomotor scanning performance in subjects with homonymous visual field disorders Visual Impairment Research 1999 1 23 31 10.1076/vimr.1.1.23.4450

[B10] Kerkhoff G Munbinger U Haaf E Eberle-Strauss G Stogerer E Rehabilitation of homonymous scotomata in patients with postgeniculate damage of the visual system: saccadic compensation training Restor Neurol Neurosci 1992 4 245 254 21551879 10.3233/RNN-1992-4402

[B11] Pambakian ALM Mannan SK Hodgson TL Kennard C Saccadic visual search training: a treatment for patients with homonymous hemianopia J Neurol Neurosurg Psychiatry 2004 75 1443 1448 10.1136/jnnp.2003.025957 15377693 PMC1738779

[B12] Zihl J Eye movement patterns in hemianopic dyslexia Brain 1995 118 891 912 10.1093/brain/118.4.891 7655887

[B13] Bouwmeester Heutink J Lucas C The effect of visual training for patients with Visual field defects due to brain damage: a systematic review J Neurol Neurosurg Psychiatry 2004 75 1443 1448 10.1136/jnnp.2003.025957 17135455 PMC2077942

[B14] Klavora P Gaskovski P Heselgrave RJ Quinn RP Young M Rehabilitation of visual skills using the Dynavision: A single case experimental study Can J Occup Ther 1995 62 37 43

[B15] Klavora P Gaskovski P Martin K Forsyth RD Heselgrave RJ Young M Quinn RP The effects of Dynavision on rehabilitation on behind-the-wheel driving ability and selected psychomotor abilities of persons after stroke Am J Occup Ther 1995 49 534 542 7645666 10.5014/ajot.49.6.534

[B16] Crotty M George S Retraining visual processing skills to improve driving ability following stroke Arch Phy Med Rehabil 2009 90 2096 2102 10.1016/j.apmr.2009.08.143 19969174

[B17] Kasten E Sabel BA Visual field enlargement after computer training in brain-damaged patients with homonymous deficits: an open pilot trial Restor Neurol Neurosci 1995 8 113 127 21551894 10.3233/RNN-1995-8302

[B18] McFadzean RM NovaVision: vision restoration therapy Curr Opin Opthalmol 2006 17 498 503 10.1097/ICU.0b013e3280108544 17065915

[B19] Teichman JC Markowitz SN Canadian research contributions to low-vision rehabilitation Can J Ophthalmol 2008 43 4 414 8 10.3129/I08-065 18711453

[B20] Vasiliou C Homonymous Hemianopia: Training compensatory strategies Australian Orthoptic Journal 1990 26 40 41

[B21] Landers J Sharma A Goldberg I Graham S A comparison of perimetric results with the Medmont and Humphrey perimeters Br J Ophthalmol 2003 87 690 4 10.1136/bjo.87.6.690 12770962 PMC1771686

[B22] Folstein MF Folstein SE McHugh PR Mini-Mental state: a practical method for grading the cognitive state of patients for the clinician J Psychiatr Res 1975 12 189 198 10.1016/0022-3956(75)90026-6 1202204

[B23] Verlander D Hayes A McInnes JK Liddle RJ Liddle GW Clarke GERussell M Ferguson W Walsh PG Assessment of clients with visual spatial disorders; a pilot study Visual impairment research 2000 2 129 142 10.1076/vimr.2.3.129.4422

[B24] Stelmack J Stelmack T Fraim M Warrington J Clinical use of the Pepper Visual Skills for Reading Test in Low Vision Rehabilitation American Journal of Optometry and Physiological Optics 2006 64 829 831 10.1097/00006324-198711000-00005 3425678

[B25] Stelmack JA Stelmack TR Massof RW Measuring Low-Vision rehabilitation Outcomes with the NEI VFQ-25 Invest Ophthalmol Visual Science 2002 43 9 12202503

[B26] Stelmack JA Szlyk JP Stelmack TR Demers-Turco P Williams RT Moran D Massof RW Measuring outcomes of vision rehabilitation with the Veterans Low Vision Visual Functioning Questionnaire Invest Ophthalmol Visual Science 2006 47 3253 61 10.1167/iovs.05-1319 16877389

[B27] Malec J The Mayo-Portland Adaptability Inventory. The Center for Outcome Measurement in Brain Injury http://tbims.org/combi/mpai/Accessed 27/2/09

[B28] Wilson Cockburn J Halligan P Development of a behavioural test of visuospatial neglect Arch Phy Med Rehabil 1987 68 98 102 3813864

[B29] Kwakkel G van Peppen R Wagenaar RC Wood Dauphinee S Richards C Ashburn A Miller K Lincoln N Partridge C Wellwood I Langhorne P Effects of augmented exercise therapy time after stroke. A metaanalysis Stroke 2004 35 2529 36 10.1161/01.STR.0000143153.76460.7d 15472114

